# Alexithymia among patients with unexplained physical symptoms

**DOI:** 10.1192/j.eurpsy.2021.670

**Published:** 2021-08-13

**Authors:** A. Rady, R. Alamrawy, I. Ramadan, M. Elmissiry

**Affiliations:** 1 Psychiatry, Alexandria University school of Medicine, Alexandria, Egypt; 2 Neurology, Alexandria University school of Medicine, alexandria, Egypt

**Keywords:** Toronto Alexithymia Scale TAS, psychosomatic, somatization, alexithymia

## Abstract

**Introduction:**

Some research suggests that mental health problems can be brought on by the stress of having unexplained symptom. In non-western cultures especially, psychological distress is often communicated through multiple somatic complaints. The biopsychosocial model takes into consideration all factors affecting health and disease, supporting the integration of biological, psychological and social factors in the assessment and treatment.

**Objectives:**

In our study we assess prevalence of alexithymia as a potential psychopathological attribute manifesting as unexplained somatic symptoms

**Methods:**

196 patients aged 18 to 60 with unexplained physical symptoms for at least three months, after collection of demographic data, medical and psychiatric history, were subject to Arabic version of the following scales : patient health questionnaire PHQ-15 to assess severity of somatic symptoms, patient health questionnaire PHQ-9 to assess depressive symptoms, generalized anaxiety disorder GAD-7 to assess general anxiety disorder symptoms and Toronto Alexithymia scale TAS to assess alexithymia

**Results:**

90% of ours ample were female patients, 49,5% showed alexithymia, 27,6% were borderline alexithymic and 23% had no alexithymia. Patients with unexplained physical symptoms showed moderate to high depressive symptoms in 81,1% of the sample, moderate to severe anxiety symptoms in 73,5%. Severity of somatic symptoms as assessed by PHQ-15 were significantly highly correlated to scores for Alexithymia (TAS), depressive symptoms (PHQ-9) and anxiety symptoms (GAD-7) p<0,001

**Conclusions:**

Alexithymia is prevalent among patients with unexplained physical symptoms. This later population has high prevalence of depressive and anxiety symptoms that go with the severity of somatic manifestations

